# P-1529. Antimicrobial resistance and virulence gene profiles of Klebsiella spp. blood isolates misidentified as Klebsiella pneumoniae

**DOI:** 10.1093/ofid/ofaf695.1710

**Published:** 2026-01-11

**Authors:** Christian Francisco, joana Marie Gantuangco, Jonnel B Poblete, Stessi Marie G Geganzo, Angelo dela Tonga, Ia Marie Donna Cruz, Sohei Harada, Masahiro Suzuki, Yohei Doi

**Affiliations:** National Institutes of Health, University of the Philippines Manila, Manila, National Capital Region, Philippines; Philippine General Hospital, Manila, National Capital Region, Philippines; Philippine General Hospital, University of the Philippines, Manila, Manila, National Capital Region, Philippines; University of the Philippines Manila, Manila, National Capital Region, Philippines; National Institutes of Health, University of the Philippines Manila, Manila, National Capital Region, Philippines; Lung Center of the Philippines, Quezon City, National Capital Region, Philippines; Toho University School of Medicine, Ota-ku, Tokyo, Japan; Fujita Health University School of Medicine, Toyoake, Aichi, Japan; Fujita Health University, Aichi, Aichi, Japan

## Abstract

**Background:**

Antimicrobial resistance (AMR) continues to pose a significant challenge in modern medicine. *Klebsiella pneumoniae* (KP) is a major cause of nosocomial infections and is frequently resistant to multiple antibiotics. Current methods in the detection of KP include the use of automated systems like Vitek 2, which rely on general biochemical characteristics of the KP complex, making it difficult to differentiate KP from closely related species like *K. variicola* and *K. quasipneumoniae.* These organisms are genetically distinct from KP and may differ in virulence characteristics and clinical features. In this study, we investigated if AMR and virulence genes found in KP are also present in *Klebsiella* spp. isolates misidentified as KP.

**Methods:**

We analyzed presumptive KP isolates from blood cultures collected from patients at the Philippine General Hospital between October 2023 and August 2024. Species identification was confirmed using polymerase chain reaction (PCR) and whole genome sequencing (WGS). The species, sequence type (ST), AMR, and virulence genes were identified using the Kleborate software

**Results:**

Among 60 unique blood isolates initially identified as KP, seven (12%) were reclassified as non-KP *Klebsiella* spp. These included *K. quasipneumoniae* (5 isolates), *K. variicola* (1 isolate), and *K. africana* (1 isolate). Notably, β-lactamase genes *bla*_NDM-7_ and *bla*_CTX-M-123_ were identified in two *K. quasipneumoniae* isolates.None of the isolates exhibited hypermucoid phenotypes or carried hypervirulence-associated genes (*iuc*, *iro*, *rmp*, *rmpA2*). Despite the absence of these virulence genes, all-cause in-hospital mortality was seen in 4 of 7 patients (57%).

**Conclusion:**

While hypervirulence-associated genes were absent in non-KP *Klebsiella* spp., infections caused by these organisms were associated with a high mortality rate. Accurate species identification of the KP complex is essential for understanding epidemiology and guiding appropriate treatment.

**Disclosures:**

Sohei Harada, MD, PhD, Denka: Honoraria|Eiken Chemical Co., Ltd.: Honoraria|MSD: Honoraria|Pfizer: Advisor/Consultant|Pfizer: Honoraria|Shionogi: Advisor/Consultant|Shionogi: Honoraria|Ushio, Inc.: Honoraria Yohei Doi, MD, PhD, GSK: Advisor/Consultant|Meiji Seika Pharma: Advisor/Consultant|Shionogi: Advisor/Consultant|Shionogi: Honoraria

